# Metabolic Syndrome and Its Associations with Components of Sarcopenia in Overweight and Obese Older Adults

**DOI:** 10.3390/jcm8020145

**Published:** 2019-01-27

**Authors:** Jakub Mesinovic, Lachlan B. McMillan, Catherine Shore-Lorenti, Barbora De Courten, Peter R. Ebeling, David Scott

**Affiliations:** 1 Department of Medicine, School of Clinical Sciences at Monash Health, Faculty of Medicine, Nursing and Health Sciences, Monash University, Clayton, VIC 3168, Australia; lachlan.mcmillan@monash.edu (L.B.M.); cat.shore-lorenti@monash.edu (C.S.-L.); peter.ebeling@monash.edu (P.R.E.); david.scott@monash.edu (D.S.); 2 Monash Centre for Health Research and Implementation, School of Public Health and Preventive Medicine, Monash University, Clayton, VIC 3168, Australia; barbora.decourten@monash.edu; 3 Australian Institute for Musculoskeletal Science (AIMSS), Department of Medicine-Western Health, Melbourne Medical School, The University of Melbourne, St Albans, VIC 3021, Australia

**Keywords:** metabolic syndrome, sarcopenia, obesity, older adults

## Abstract

Ageing, obesity and the metabolic syndrome (MetS) may all contribute to poor muscle health (sarcopenia). This study aimed to determine the cross-sectional associations between MetS (International Diabetes Federation classification) and sarcopenia (revised European Working Group on Sarcopenia in Older People definition) in 84 overweight and obese older adults. Components of sarcopenia included muscle strength (hand grip and leg extension), physical performance (stair climb test and short physical performance battery (SPPB), including gait speed and repeated chair stands time), muscle mass (appendicular lean mass (ALM), dual-energy X-ray absorptiometry), muscle size (peripheral quantitative computed tomography-determined calf and forearm cross-sectional area (CSA)) and muscle quality (muscle density and strength normalised to lean mass). Waist circumference was associated with greater muscle size, but poorer leg extension strength, chair stands and stair climb time, gait speed, SPPB scores and muscle quality measures (all *p* < 0.05). MetS was positively associated with ALM and forearm muscle CSA, and negatively associated with muscle quality measures and chair stands time (all *p* < 0.05). MetS is associated with larger muscle size, yet poorer muscle quality in overweight and obese older adults. Assessments of muscle function and quality should be considered for obese older adults and those with MetS.

## 1. Introduction

The metabolic syndrome (MetS) is a cluster of risk factors that collectively and independently increase the risk of developing type 2 diabetes mellitus (T2DM) and cardiovascular disease (CVD) [1]. These risk factors include central obesity, raised glucose and triglyceride concentrations, low high-density lipoprotein (HDL) concentrations and raised blood pressure [2].

The prevalence of MetS increases with age [3] and adiposity [4]. All of these factors are associated with an increased falls risk [5,6,7]. MetS may affect muscle health through various mechanisms. Briefly, central adiposity is associated with increased systemic proinflammatory cytokine concentrations [8]. Impaired glycaemic control can result in peripheral neuropathy [9], myopathy [10] and inflammation [11], and both dyslipidaemia and hypertension can lead to micro- and macro-vascular complications [12,13]. Inflammation, neuropathy, myopathy and vascular complications are negatively associated with muscle strength, mass, size and quality, which are components of sarcopenia [14,15,16,17,18,19,20].

Sarcopenia is a term used to describe the age-related decline in muscle mass, strength and physical performance [21]. A recent meta-analysis by Zhang et al. reported that non-obese middle-aged and older adults with sarcopenia have two-fold higher odds of having MetS compared to healthy controls [22]. This may be attributed, at least in part, to the important role skeletal muscle plays in the metabolism of macronutrients including glucose and lipids. Interestingly, The European Working Group for Sarcopenia (EWGSOP) recently updated their definition of sarcopenia (EWGSOP2) and stated that muscle quality is a research gap that requires further investigation [23]. Muscle quality is a term used to describe muscle composition and function per unit of muscle mass [24]. Measures of muscle quality have been reported to be associated with physical function, falls and markers of metabolic health [25,26,27,28,29]. Currently, there is no consensus definition for measures of muscle quality. However, proposed measures of muscle quality include inter- and intra-muscular adipose tissue (IMAT) and/or muscle density (an indirect measure of IMAT) [24], and also strength relative to lean mass [30], which can be quantified using various imaging modalities [31,32]. Associations between MetS and these muscle quality parameters are presently unclear.

This study aimed to determine the associations between MetS and components of sarcopenia, including muscle mass and quality, absolute and relative strength, and also physical performance, in overweight and obese older adults.

## 2. Methods

### 2.1. Study Design and Participants

Eighty-four overweight or obese (body mass index (BMI ≥ 25kg/m^2^)) community-dwelling older (aged ≥50 years) men and women residing in Melbourne, Australia were recruited for this study. Participants responded to print and online advertisements, were ambulant, English speaking and had no self-reported diagnosis of psychotic or progressive neurological disorders, severe arthritis (awaiting a joint replacement) or life expectancy <12 months. This study was approved by the Melbourne Health Human Research Ethics Committee (HREC 2013.294) and participants provided written informed consent. All testing was conducted at the Clinical Trials Unit at the Australian Institute for Musculoskeletal Science (AIMSS) between March 2014 and August 2016.

### 2.2. Questionnaire

A self-administered questionnaire included questions on demographics, chronic health conditions (including diabetes), medication use and weekly physical activity levels. Total minutes of weekly moderate and vigorous physical activity (MVPA) were calculated using the Active Australia Survey [33].

### 2.3. Physical Function

Hand grip strength of the dominant hand was assessed using a Jamar Plus Digital hydraulic hand grip dynamometer (Patterson Medical, Bolingbrook, IL, USA) [34]. Participants were seated with their arm fully extended at shoulder height, parallel with the ground and gripped the dynamometer with maximal force for three seconds. The test was completed three times, with a 30 s rest between trials, and the mean maximal force was calculated. Leg extension strength was assessed using a hand-held dynamometer (HHD; Lafayette Manual Muscle Tester Model 01165, Lafayette Instrument Company, Lafayette, IN, USA). The participant was seated with their arms folded across their chest, hip and knee joint angles at 90°, and feet above the floor. The participant exerted maximal force for three seconds to the HHD, which was held stationary by the tester, about 10 cm above the ankle joint [35]. Mean peak force from three trials was calculated.

Participants completed a short physical performance battery (SPPB), which is a validated measure of physical performance and disability in older adults [36]. A summary score of 0–12 (higher score indicating better function) was obtained based on performance in three tasks: repeated chair stands, standing balance assessments and gait speed over a 2.44 m course. Participants with SPPB scores ≤9 (low SPPB score) were considered to have poor physical performance [37].

Mean stair climb time was measured by two attempts at ascending a 10 step flight of stairs as quickly as possible (participants could use the handrail if required) [38].

### 2.4. Anthropometry, Body Composition and Muscle Quality

Height (Seca 222 wall-mounted stadiometer, Seca, Hamburg, Germany) and weight (Seca 804 electronic scales, Seca, Hamburg, Germany) were measured without footwear and heavy items of clothing. Height and weight were used to calculate body mass index (BMI: weight (kg)/ height (m^2^)).

Waist circumference was measured twice, to the nearest 0.1 cm, using a measuring tape (Seca 203) at the mid-point between the inferior margin of the last rib and the crest of the ilium in the mid-axillary plane. If there was a difference of more than 2 cm between readings 1 and 2, a third measurement was performed. The average of these readings was then calculated.

Total body fat percentage, appendicular lean mass (ALM; kg) and visceral adipose tissue (VAT) area (cm^2^) were measured using dual energy x-ray absorptiometry (DXA) (Hologic Discovery W, Hologic, Bedford, MA, USA). The study DXA was calibrated daily using the manufacturer’s spine phantom, and the short-term intra-individual coefficient of variation (CV) for ALM was 1.0%.

Upper-limb relative strength was calculated using the following equation: average hand grip strength in dominant arm (kg) divided by lean mass (determined by DXA) in the corresponding limb (kg). Lower-limb relative strength was calculated using: average leg extension strength in dominant leg (kg) divided by lean mass (determined by DXA) in the corresponding limb (kg) [30].

A single 2.5 mm transverse peripheral quantitative computed tomography (pQCT) (Stratec XCT3000, Stratec Medizintechnik GmbH, Pforzheim, Germany) scan (speed of 20 mm/s and voxel size of 0.8 mm) was obtained at 66% of radial and tibial length of the non-dominant arm and dominant leg, respectively. The length of the radius was determined by distance between the distal end of the ulna styloid and the tip of the olecranon. The length of the tibia was determined by distance between the prominence of the medial malleolus and the tibial plateau. A planar scout view of the distal radius and tibia was used to locate scan sites and reference lines were placed, bisecting the medial border of the distal radial joint surface and parallel to the distal joint surface of the tibia. The dominant leg was preferentially selected for this assessment in order to allow comparability of muscle composition measures, with leg extension strength assessed in the same limb. All pQCT scans were acquired and analysed by one observer. Forearm and calf muscle cross-sectional area (CSA) (mm^2^) and density (mg/cm^3^) (density of tissue within the muscle compartment after removal of subcutaneous fat and bone areas) were determined using manufacturer’s algorithms and software (version 6.2). The device was calibrated daily using the manufacturer’s phantom and the short-term intra-individual CV for muscle density was 1.0%.

### 2.5. Cardiometabolic Measures

Blood pressure was measured using a digital sphygmomanometer (Welch Allyn Connex Pro BP 3400, Welch Allyn, NY, USA). The arm cuff was placed at least 3 cm above the elbow joint and inflated by starting the device. Once fully deflated, systolic (SBP) and diastolic blood pressure (DBP) were recorded. A second measurement was obtained after a 30 s break and a third reading was taken if there was a difference of more than 25 mmHg for SBP, or 15 mmHg for DBP, between the first two readings.

A blood sample was collected after an overnight fast of at least 10 h. Serum glucose, HDL and triglyceride concentrations were analysed using the automated ADVIA 1650 Chemistry System (Siemens Healthcare Diagnostics Incorporation, Victoria, Australia). One participant did not have glucose or insulin analysed.

### 2.6. MetS Definition

MetS was defined using the International Diabetes Federation (IDF) criteria [2]. MetS classification using this definition requires central obesity (defined as elevated waist circumference according to ethnicity specific cut-points) and two or more of the following factors: raised fasting glucose (≥5.6 mmol/L or previously diagnosed diabetes and reported use of glucose-lowering drugs), raised triglycerides (≥1.7mmol/L or reported use of triglyceride-lowering drugs), HDL cholesterol (<1.03 mmol/L in men and <1.29 mmol/L in women, or reported use of drugs that increase HDL concentrations), raised blood pressure (SBP ≥130 or DBP ≥85 mmHg or reported use of antihypertensive drugs) [2].

### 2.7. Sarcopenia Definition

Sarcopenia was defined using the EWGSOP2 criteria. Participants were considered to have probable sarcopenia if they had: grip strength <27 kg (men) or <16 kg (women) or chair stands time >15 s for 5 rises. Confirmed sarcopenia was defined as: probable sarcopenia plus ALM <20 kg (men) or <15 kg (women) or ALM/height^2^ <7 kg/m^2^ (men) or <6 kg/m^2^ (women). Severe sarcopenia was defined as: confirmed sarcopenia plus gait speed ≤0.8 m/s or SPPB score ≤8 [23].

### 2.8. Statistical Analyses

Continuous data were assessed for normality using boxplots and Shapiro-Wilk tests. Independent samples t-tests and Mann-Whitney U tests compared continuous variables, and Chi-square tests compared categorical variables between individuals with and without MetS. Pearson’s correlations examined associations between components of sarcopenia alone, and with components of MetS. Multivariable linear regression analyses explored associations between components of MetS and sarcopenia, and was adjusted for age, gender and body fat percentage. Multivariable linear regression analyses also explored associations between MetS and components of sarcopenia, and were adjusted for age and gender. Variance inflation factors were examined to inspect for multicollinearity in all models and tolerance was set at 10. *p*-values <0.05 or 95% confidence intervals (CI) not including the null point were considered statistically significant. All analyses were performed in SPSS Statistics version 25 (IBM, Armonk, NY, USA).

## 3. Results

The median BMI in this cohort was 30.4 (IQR: 27.2, 36.3) kg/m^2^ and 52% of participants were obese (BMI ≥30 kg/m^2^). Probable, confirmed and severe sarcopenia prevalence according to the EWGSOP2 algorithm were 35%, 0% and 2%, respectively. Table 1 presents descriptive characteristics according to MetS status. Individuals with MetS had higher BMI, VAT, ALM and prevalence of self-reported diabetes. Individuals without MetS had higher upper- and lower-limb relative strength and forearm and calf muscle density. There were no differences in age, gender distribution and MVPA between individuals with and without MetS.

Table 2 presents Pearson’s correlation coefficients for components of sarcopenia. In this cohort, gait speed had a small negative association with chair stands time and small positive association with forearm muscle density. Stair climb time had a moderate positive association with chair stands time and moderate negative association with forearm muscle density. Stair climb time also had small negative associations with leg extension strength, upper- and lower-limb relative strength and calf muscle density. SPPB score had a large negative association with chair stands time and small positive associations with leg extension strength, upper- and lower-limb relative strength and calf muscle density.

Pearson’s correlation coefficients for components of MetS and sarcopenia are presented in Table 3. Fasting glucose had a small positive association with ALM and small negative associations with upper- and lower-limb relative strength, forearm muscle density and SPPB score. Waist circumference had small positive associations with hand grip strength, stair climb time and forearm muscle CSA. Waist circumference also had a moderate positive association with calf muscle CSA and ALM. Waist circumference had small negative associations with upper- and lower-limb relative strength, forearm and calf muscle density, gait speed and SPPB score. Triglycerides had small negative associations with leg extension strength and lower-limb relative strength. HDL cholesterol had small negative associations with hand grip strength and forearm and calf muscle CSA and a small positive association with lower-limb relative strength. HDL cholesterol also had a moderate negative association with ALM. Systolic blood pressure had a small negative association with calf muscle density.

Table 4 presents findings from multivariable linear regression between components of MetS and sarcopenia. Individuals with raised fasting glucose or self-reported diabetes had poorer leg extension strength, forearm muscle density and lower-limb relative strength. Waist circumference was positively associated with chair stands and stair climb time, forearm and calf muscle CSA, gait speed and ALM. Waist circumference was also negatively associated with upper- and lower-limb relative strength, forearm and calf muscle density and leg extension strength. Individuals with raised triglycerides or reported use of triglyceride-lowering drugs had poorer leg extension strength and lower-limb relative strength. Individuals with low HDL or reported use of drugs that increase HDL concentrations had poorer upper- and lower-limb relative strength, leg extension strength and higher ALM. Individuals with raised blood pressure or reported use of antihypertensive medication had higher ALM. A sensitivity analysis was also performed for raised fasting glucose, triglycerides and blood pressure and low HDL, which was adjusted for age, gender and waist circumference (data not shown). All associations between raised fasting glucose and components of sarcopenia became non-significant. Previous associations between raised tryglycerides and components of sarcopenia remained significant and a negative association with ALM became significant. The negative association between low HDL and upper-limb relative strength remained significant and all other previous associations became non-significant. Additionally, a negative association between low HDL and hand grip strength became significant. The association between raised blood pressure and ALM became non-significant.

Multivariable linear regression, exploring associations between MetS and sarcopenia, is presented in Table 5. Participants with MetS had lower chair stands time, upper- and lower-limb relative strength and forearm and calf muscle density, but had higher forearm muscle CSA and ALM.

## 4. Discussion

This cross-sectional study of community-dwelling, overweight and obese older adults demonstrated that MetS is associated with greater lean mass and forearm muscle size, but poorer muscle quality. These findings suggest that the greater muscle mass of overweight and obese older adults with MetS compared to those without MetS does not confer a functional advantage, and that assessments of muscle quality may be useful in identifying individuals with MetS who are at risk for functional decline.

Given that participants with MetS had a higher waist circumference, which is a strong predictor of muscle quality, it is unsurprising that these individuals had lower values for this component of sarcopenia. When we adjusted for age, gender and body fat percentage, raised fasting glucose and low HDL were negatively associated with a number of muscle quality parameters; however, these associations became non-significant in our sensitivity analysis that adjusted for waist circumference instead of body fat percentage. This suggests that perhaps VAT, which is indirectly measured through waist circumference, is negatively influencing muscle quality in individuals with MetS. Indeed, associations between VAT and IMAT have been reported in other research studies [39]. Increased IMAT/lower muscle density is likely to be influencing the negative associations between MetS and relative strength measures, as non-contractile tissue within muscle can reduce the amount of force produced per unit of muscle [40]. Interestingly, calf muscle density has also been reported to be negatively associated with falls risk in community-dwelling older adults [29,41]; however, it is unclear whether lower calf muscle density translates to increased falls risk in overweight and obese older adults with MetS. We have previously reported that higher calf muscle density independently predicts better physical function, in this cohort [42], and Liao et al. reported that MetS and all of its components (except for low HDL) are associated with increased falls risk in community-dwelling older adults [5]. Future studies need to establish whether increased falls risk in MetS occurs due its components (particularly central adiposity) and/or poorer muscle quality, and whether their co-existence has synergistic effects on falls risk.

To the best of our knowledge, only one study has prospectively determined the relationship between relative strength and falls risk, and they reported that in community-dwelling older women, low relative strength was associated with a higher incidence of falls [26]. The aforementioned study by Gadelha et al. calculated relative strength using a ratio of strength to muscle CSA; therefore, it is unclear whether low relative strength measured by muscle strength to mass ratio (which was used in our study) also increases falls risk. However, given the strong correlation between muscle mass and size, we hypothesize that low relative strength measured by strength to mass ratio also has the capacity to predict falls risk. It should be noted that although relative strength is predictive of physical function, factors such as adiposity, age and ALM have been reported to influence this relationship and must be taken into account when assessing the relationship between relative strength and physical function [28].

A large discrepancy was evident between individuals in this cohort with probable (34%), confirmed (0%) and severe (2%) sarcopenia. The EWGSOP2 definition now considers muscle strength the principal determinant of sarcopenia, which allows for prompt and cost-effective identification of muscle health deterioration in a clinical setting. In our cohort, 9% of individuals had low hand grip strength, whilst 42% had low chair stands time (data not shown), meaning that a large proportion of individuals had normal upper-limb strength, but poor lower-limb strength. The addition of chair stands time cut-points to the EWGSOP2 recommendations may therefore allow for identification and potential treatment of individuals at risk of disability [43], who previously may have had normal muscle strength according to upper-limb strength measures. Additionally, the EWGSOP2 recommendations now suggest assessing causes of sarcopenia and initiating interventions in individuals with probable, confirmed and severe sarcopenia, which may reduce both the incidence and prevalence of sarcopenia [23]. The discrepancy between probable and confirmed or severe sarcopenia prevalence in this study may be partly explained by the fact that muscle mass was high in this overweight and obese population, and the lack of muscle quality cut-points currently included the EWGSOP2 sarcopenia definition. Individuals with poorer muscle quality but higher muscle size, such as individuals with MetS in our cohort may therefore be improperly diagnosed, highlighting the need for muscle quality cut-points and a consensus definition.

All components of MetS, except for elevated blood pressure, were negatively associated with leg extension strength, however this did not translate to individuals with MetS having lower leg extension strength. In fact, individuals with MetS had lower chair stands time, indicating better lower-limb strength compared to individuals without MetS. Increased lower-limb strength may be partially attributed to increased muscle mass; however, despite being predictive of physical performance, muscle mass has been reported to explain as little as 13% of the variance in muscle strength in older adults, after adjusting for age and sex [44]. Increased inflammation, which is common in individuals with MetS, is typically associated with poor muscle strength. Findings from the Health ABC Study and the English Longitudinal Study of Ageing both found significant associations between elevated interleukin-6 (IL-6) concentrations and reduced grip strength, as well as negative associations between C-reactive protein and chair stands time [17,18]. IMAT is also thought to impair muscle strength through localised inflammation [45] and has been reported to be positively associated with systemic and intramuscular IL-6 concentrations in older adults [45,46]. Nevertheless, despite individuals with MetS having higher lower-limb strength compared to individuals without MetS, relative strength in both the upper- and lower-limbs remained lower.

Absolute muscle mass increases with body mass; however, percentage of muscle mass does not [47]. As mentioned previously, skeletal muscle is important for metabolising glucose and lipids and; therefore, reductions in muscle mass also have the potential to influence metabolic health. Indeed, low muscle mass has been associated with increased risk of incident T2DM [48], and T2DM has also been reported to be associated with the loss of skeletal muscle mass [49]. Sarcopenia has been proposed to be both a cause and consequence of impaired glucose metabolism [50]. We reported positive associations between MetS and muscle mass; however, we attribute these findings to the higher overall body mass in individuals with MetS. Given the cross-sectional design of our study, it is unclear whether poor metabolic health influences muscle mass, or whether muscle mass is influencing metabolic health. Despite increased muscle mass and size, individuals with MetS did not have better physical performance compared to individuals without MetS.

Physical performance is a multidimensional component of sarcopenia that involves interplay between nerves, balance centres and multiple muscle groups [51]. Therefore, a level of redundancy exists whereby a multitude of components involved in physical performance need to be impaired before it translates to poor overall physical performance [23]. This cohort was relatively young and, as such, it is possible that longitudinal studies or older cohorts would demonstrate associations between MetS and physical performance. Additionally, the relatively low prevalence of severe sarcopenia in this cohort suggests that, despite individuals with MetS having poorer muscle quality and lower-limb strength compared to individuals without MetS, this is yet to translate to clinically-significant physical performance declines in most of these individuals.

This study had a cross-sectional design, which limits comments on causation. Additionally, the cohort constituted of a small sample of generally-healthy community-dwelling older adults and, as such, it is likely the findings cannot be generalised to the wider older adult population. Given the diverse community residing in this geographical location it is possible that this cohort had high heterogeneity. Future studies would be improved by utilising gold-standard measures of IMAT, derived through magnetic resonance imaging, which may provide further insight into muscle composition in this population [52]. Additionally, the effects of medications such as glucose-lowering drugs, statins and antihypertensives on components of sarcopenia, particularly muscle quality, should be explored further in larger overweight and obese older adult populations.

In conclusion, MetS was negatively associated with all measures of muscle quality and positively associated with muscle mass and size. This suggests that increased muscle mass and size do not necessarily translate to increased quality or better physical performance in overweight and obese older adults with MetS, and more comprehensive assessments of muscle health may be warranted in this population.

## Figures and Tables

**Table 1:** Descriptive characteristics for individuals with and without metabolic syndrome.

	MetS (*N* = 64)	No MetS (*N* = 20)	*p*-Value
Age (y)	63 ± 7.1	60.3 ± 9.9	0.184
Weight (kg)	90.9 ± 19.2	79.9 ± 11.8	**0.018**
BMI (kg/m^2^)	32.5 (28.1, 38.0)	28.3 (26.3, 30)	**0.003**
Height (cm)	164.7 ± 9.8	165.7 ± 8.1	0.684
Women (%)	53	60	0.590
Sarcopenia (EWGSOP2) (%)			
Probable Sarcopenia	36	30	0.626
Confirmed Sarcopenia	0	0	
Severe Sarcopenia	2	5	0.379
Visceral Adipose Tissue (mm^2^)	220.2 ± 80.5	143.6 ± 39.4	**<0.001**
Moderate and Vigorous Physical Activity (mins/week)	30 (0, 146.3)	55 (1.3, 214)	0.294
Self-Reported Diabetes (%)	28	0	**0.007**
Medications (%)			
Glucose-lowering drugs	25	0	**0.013**
*Metformin*	22	0	**0.022**
*Gliclazide*	11	0	0.122
*Insulin*	6	0	0.252
Statins	53	0	**<0.001**
Fibrates	3	0	0.424
Antihypertensives	61	10	**<0.001**
Glucose (mmol/L) #	5.7 (5.3, 7) n = 63	5.2 (5, 5.4)	**<0.001**
Triglycerides (mmol/L) #	1.4 (1, 2)	1 (0.8, 1.5)	**0.009**
HDL (mmol/L) #	1.3 (1, 1.5)	1.6 (1.3, 1.9)	**0.002**
Waist Circumference (cm)	109.4 ± 14.2	98 ± 7.8	**<0.001**
Systolic Blood Pressure (mmHg)	141.1 ± 16.9	126.1 ± 17.3	**0.001**
Diastolic Blood Pressure (mmHg)	82.2 ± 12.0	79.8 ± 9.3	0.416
**Muscle Strength**			
Hand Grip Strength (kg)	30.9 ± 11.0	30.4 ± 9.9	0.856
Leg Extension Strength (kg)	16.5 ± 9.7	19.6 ± 6.9	0.129
Chair Stands Time (s)	13.7 ± 5.8	17.3 ± 15	0.302
**Muscle Quality**			
Upper-Limb Relative Strength (kg/kg)	10.4 ± 2.7	12 ± 2.4	**0.021**
Forearm Muscle Density (mg/cm^3^)	73 ± 5.0	76.6 ± 3.3	**0.004**
Lower-Limb Relative Strength (kg/kg)	1.9 ± 1.1	2.5 ± 0.9	**0.020**
Calf Muscle Density (mg/cm^3^)	71.4 ± 4.1	73.7 ± 2.6	**0.020**
**Muscle Size**			
Forearm Muscle CSA (cm^2^)	354.2 ± 100.1	314.0 ± 72.7	0.058
Calf Muscle CSA (cm^2^)	764.1 ± 170.6	711.3 ± 117.1	0.125
ALM (kg)	23.7 ± 6.1	21.1 ± 4.1	**0.033**
**Physical Performance**			
Gait Speed (m/s)	3.2 ± 0.8	0.7 ± 0.2	0.125
Stair Climb Time (s)	5.9 ± 2.7	5.8 ± 2.1	0.810
SPPB score	10 (9, 11)	10 (8, 12)	0.753

All data are mean ±SD or frequency (%) unless otherwise specified. # median (IQR). MetS-metabolic syndrome; BMI-body mass index; EWGSOP2- Updated European Working Group on Sarcopenia in Older People definition; HDL-high-density lipoprotein; CSA-cross-sectional area; ALM-appendicular lean mass; SPPB-short physical performance battery. *p* < 0.05.

**Table 2:** Pearson’s correlation coefficients (*p*-values) for measures of muscle strength, quality and size with physical performance.

	Gait Speed (m/s)	Stair Climb Time (s)	SPPB Score #
**Muscle Strength**			
Hand Grip Strength (kg)	0.02 (0.878)	−0.17 (0.125)	0.10 (0.352)
Leg Extension Strength (kg)	0.16 (0.149)	**−0.36** **(0.001)**	**0.32** **(0.004)**
Chair Stands Time (s)	**−0.40** **(<0.001)**	**0.57** **(<0.001)**	**−0.82** **(<0.001)**
**Muscle Quality**			
Upper-Limb Relative Strength (kg/kg)	0.14 (0.209)	**−0.28** **(0.010)**	**0.27** **(0.013)**
Forearm Muscle Density (mg/cm^3^)	**0.29** **(0.009)**	**−0.51** **(<0.001)**	0.17 (0.130)
Lower-Limb Relative Strength (kg/kg)	0.15 (0.175)	**−0.35** **(0.001)**	**0.31** **(0.004)**
Calf Muscle Density (mg/cm^3^)	0.18 (0.111)	**−0.43** **(<0.001)**	**0.25** **(0.021)**
**Muscle Size**			
Forearm Muscle CSA (cm^2^)	−0.09 (0.407)	0.02 (0.849)	−0.04 (0.748)
Calf Muscle CSA (cm^2^)	−0.03 (0.791)	0.03 (0.811)	−0.11 (0.315)
ALM (kg)	−0.08 (0.475)	0.18 (0.107)	−0.11 (0.306)

# Spearman’s correlations. SPPB-short physical performance battery; CSA-cross-sectional area; ALM-appendicular lean mass. *p* < 0.05.

**Table 3:** Pearson’s correlation coefficients (*p*-values) for components of metabolic syndrome and sarcopenia.

	Glucose (mmol/L) # *N* = 83	Waist Circumference (cm)	Triglycerides (mmol/L) #	HDL Cholesterol (mmol/L) #	Systolic Blood Pressure (mmHg)	Diastolic Blood Pressure (mmHg)
**Muscle Strength**						
Hand Grip Strength (kg)	−0.05 (0.630)	**0.28** **(0.009)**	0.06 (0.600)	**−0.37** **(0.001)**	0.10 (0.381)	0.07 (0.535)
Leg Extension Strength (kg)	−0.19 (0.084)	−0.14 (0.212)	**−0.23** **(0.040)**	0.03 (0.781)	0.10 (0.359)	0.03 (0.761)
Chair Stands Time (s)	0.20 (0.070)	0.22 (0.052)	−0.09 (0.429)	−0.04 (0.727)	−0.04 (0.725)	−0.16 (0.146)
**Muscle Quality**						
Upper-Limb Relative Strength (kg/kg)	**−0.24** **(0.028)**	**−0.26** **(0.017)**	−0.03 (0.818)	0.04 (0.708)	−0.09 (0.432)	0.10 (0.392)
Forearm Muscle Density (mg/cm^3^)	**−0.27** **(0.014)**	**−0.44** **(<0.001)**	−0.10 (0.362)	−0.08 (0.462)	−0.18 (0.114)	0.03 (0.819)
Lower-Limb Relative Strength (kg/kg)	**−0.25** **(0.022)**	**−0.41** **(<0.001)**	**−0.29** **(0.009)**	**0.23** **(0.038)**	0.04 (0.745)	0.02 (0.856)
Calf Muscle Density (mg/cm^3^)	−0.16 (0.140)	**−0.43** **(<0.001)**	0.08 (0.495)	−0.01 (0.902)	**−0.28** **(0.010)**	0.001 (0.999)
**Muscle Size**						
Forearm Muscle CSA (cm^2^)	0.06 (0.583)	**0.49** **(<0.001)**	0.06 (0.567)	**−0.48** **(<0.001)**	0.13 (0.255)	−0.05 (0.644)
Calf Muscle CSA (cm^2^)	0.11 (0.334)	**0.57** **(<0.001)**	0.08 (0.472)	**−0.47** **(<0.001)**	0.05 (0.628)	0.04 (0.744)
ALM (kg)	**0.23** **(0.033)**	**0.69** **(<0.001)**	0.08 (0.500)	**−0.52** **(<0.001)**	0.11 (0.308)	0.01 (0.914)
**Physical Performance**						
Gait Speed (m/s)	−0.16 (0.158)	**−0.22** **(0.043)**	0.16 (0.152)	−0.02 (0.876)	0.12 (0.271)	0.08 (0.474)
Stair Climb Time (s)	0.16 (0.143)	**0.41** **(<0.001)**	−0.06 (0.574)	0.06 (0.611)	0.03 (0.805)	−0.09 (0.393)
SPPB Score #	**−0.28** **(0.012)**	**−0.27** **(0.013)**	0.07 (0.514)	0.05 (0.663)	0.06 (0.581)	0.04 (0.711)

# Spearman’s correlations. CSA-cross-sectional area; ALM-appendicular lean mass; SPPB-short physical performance battery. *p* < 0.05.

**Table 4:** Multivariable regression exploring associations between components of metabolic syndrome and sarcopenia.

	Raised Fasting Glucose *N* = 83	Waist Circumference (cm)#+	Raised Triglycerides	Low HDL	Raised Blood Pressure
**Muscle Strength**					
Hand Grip Strength (kg)	0.2 (−2.8, 3.2)	0.1 (−0.1, 0.2)	−1.2 (−4.7, 2.2)	−3.6 (−7.6, 0.4)	0.8 (−3.0, 4.7)
Leg Extension Strength (kg)	**−4.1** **(−7.8, -0.3)**	**−0.2** **(−0.3, −0.04)**	**−5.6** **(−9.8, −1.3)**	**−5.4** **(−10.4, −0.3)**	0.7 (−4.4, 5.7)
Chair Stands Time (s)	−0.3 (−4.2, 3.6)	**0.2** **(0.03, 0.3)**	0.2 (−4.4, 4.7)	1.9 (−3.4, 7.3)	−0.8 (−5.7, 4.1)
**Muscle Quality**					
Upper-Limb Relative Strength (kg/kg)#	−0.9 (−2.1, 0.2)	**−0.1** **(−0.1, −0.02)**	−0.5 (−1.8, 0.9)	**−2.1** **(−3.6, −0.6)**	−0.7 (−2.2, 0.8)
Forearm Muscle Density (mg/cm^3^)	**−2.4** **(−4.5, −0.2)**	**−0.2** **(−0.3, −0.1)**	−1.5 (−4.0, 1.0)	−2.2 (−5.1, 0.8)	−2.5 (−5.2, 0.1)
Lower-Limb Relative Strength (kg/kg)#	**−0.6** **(−1.1, −0.2)**	**−0.04** **(−0.1, −0.02)**	**−0.7** **(−1.2, −0.2)**	**−0.8** **(−1.4, −0.1)**	−0.2 (−0.8, 0.5)
Calf Muscle Density (mg/cm^3^)	−1.2 (−2.9, 0.5)	**−0.1** **(−0.2, −0.1)**	−0.2 (−2.2, 1.7)	−2.0 (−4.2, 0.3)	−1.7 (−3.8, 0.4)
**Muscle Size**					
Forearm Muscle CSA (cm^2^)	19.2 (−3.6, 41.9)	**1.9** **(1.3, 2.7)**	3.6 (−23.3, 30.5)	12.5 (−19.5, 44.5)	15.8 (−13.0, 44.7)
Calf Muscle CSA (cm^2^)	32.3 (−21.2, 85.8)	**5.0** **(3.3, 6.7)**	−7.3 (−69.9, 55.4)	58.0 (−14.7, 130.7)	58.4 (−8.5, 125.2)
ALM (kg)#	1.71 (−0.1, 3.5)	**0.2** **(0.2, 0.3)**	−0.6 (−2.7, 1.5)	**3.0** **(0.6, 5.4)**	**2.6** **(0.3, 4.8)**
**Physical Performance**					
Gait Speed (m/s)	−0.03 (−0.1, 0.04)	**−0.003** **(−0.006, −0.0001)**	0.01 (−0.1, 0.1)	−0.01 (−0.1, 0.1)	0.1 (−0.04, 0.2)
Stair Climb Time (s)	0.4 (−0.7, 1.6)	**0.1** **(0.1, 0.1)**	0.5 (−0.8, 1.8)	1.0 (−0.6, 2.5)	0.4 (−1.0, 1.8)

Data are unstandardised beta-coefficients (95% confidence intervals) adjusted for age, gender and body fat percentage. # Adjusted for age and gender. + Continuous variable. CSA-cross-sectional area; ALM-appendicular lean mass. *p* < 0.05.

**Table 5:** Multivariable regression exploring associations between metabolic syndrome and components of sarcopenia.

	β-Coefficient (95% CI)
**Muscle Strength**	
Hand Grip Strength (kg)	−0.3 (−4.0, 3.3)
Leg Extension Strength (kg)	−4.0 (−8.7, 0.6)
Chair Stands Time (s)	**−4.7 (−9.1, −0.3)**
**Muscle Quality**	
Upper-Limb Relative Strength (kg/kg)	**−1.5 (−2.9, −0.2)**
Forearm Muscle Density (mg/cm^3^)	**−3.5 (−5.9, −1.1)**
Lower-Limb Relative Strength (kg/kg)	**−0.7 (−1.3, −0.2)**
Calf Muscle Density (mg/cm^3^)	**−2.1 (−4.0, −0.1)**
**Muscle Size**	
Forearm Muscle CSA (mm^2^)	**30.5 (4.3, 56.7)**
Calf Muscle CSA (cm^2^)	46.1 (−16.6, 108.9)
Appendicular Lean Mass (kg)	**2.5 (0.4, 4.5)**
**Physical Performance**	
Gait Speed (m/s)	0.1 (−0.02, 0.2)
Stair Climb Time (s)	0.1 (−1.3, 1.4)
Low SPPB score #	0.7 (0.3, 2.1)

Data are unstandardised beta-coefficients (95% confidence intervals) adjusted for age and gender. CSA-cross-sectional area; SPPB-short physical performance battery. # Odds ratio. *p* < 0.05.
